# A light-weight symptom checker and its methodological validation

**DOI:** 10.1007/s11259-026-11263-8

**Published:** 2026-05-25

**Authors:** Welf Löwe, Gisa Löwe

**Affiliations:** 1 Linnaeus Center for Data Intensive Sciences and Applications, Linnaeus University, Växjö/Kalmar, Sweden; 2 Tierklinik Berlin-Marzahn, Berlin, Germany

**Keywords:** Symptom checker, Diagnostic support system, Validation, Companion animals, Synthetic vignettes

## Abstract

**Introduction:**

Early recognition of diseases in pets is essential, yet owners often face challenges in interpreting clinical symptoms. Digital symptom checkers offer a promising approach to encode veterinary knowledge, but their reliability and diagnostic accuracy remain largely unvalidated. This study addresses this gap through a method validation of a expert-knowledge-based veterinary symptom checker using synthetically generated test cases, enabling systematic exploration of the symptom–disease space in the absence of clinical data.

**Methods:**

System performance was quantified using simulated user–checker dialogs across $\boldsymbol{\approx }$550 diseases for dogs and cats, respectively. Robustness and efficiency were evaluated through three research questions: convergence probability, convergence speed, and structural factors influencing convergence.

**Results:**

The system achieved full convergence under ideal conditions (100%), with rapid convergence (mean rank of one after $\boldsymbol{\approx }$20 questions) and short response times (0.213–0.258 msec per disease). Under probabilistic user-answering strategies, performance decreased slightly but remained robust, with non-converging cases rare and correct diagnoses typically among top-ranked results (ranks 1–6 for dogs; 1–4 for cats). Structural analysis identified the number and uniqueness of symptoms as key predictors of diagnostic difficulty, with significant variation across anatomical regions.

**Discussion:**

Findings confirm the system’s internal consistency, robustness, and computational efficiency, establishing a validated foundation for evidence-based veterinary diagnostic support. Future work will include clinical and user studies to confirm performance under authentic conditions and address current limitations of synthetic data.

## Introduction

Early recognition of diseases in companion animals is essential for effective treatment, for reducing animal suffering, and for the ease of mind of their owners. However, pet owners often face difficulties in assessing the severity or relevance of observed symptoms, which can delay seeking veterinary care or lead to unnecessary visits. Digital symptom checkers, well established in human healthcare, offer a means to support initial triage, provide educational feedback, and promote timely veterinary consultation. Despite their potential, few systems exist that are specifically designed and validated for animals, and their reliability and accuracy remain largely unexamined.

While expert-based symptom checkers can encode valuable veterinary knowledge in a structured and accessible form, their practical usefulness depends on the correctness and internal consistency of the underlying rules. Without systematic validation, even minor inconsistencies or gaps in the expert knowledge base may lead to misleading suggestions or reduced trust among users and professionals. Direct validation against real clinical data is challenging due to limited case availability and the heterogeneity of symptom reporting by pet owners. Therefore, this study performs a method validation using synthetically generated test cases that systematically explore the symptom–disease space, not to substitute clinical trials and user studies, but rather to systematically improve and stabilize the expert knowledge database and the symptom checker inference algorithm prior to these more expensive validations and large-scale deployment.

To assess the reliability and behavior of the symptom checker under controlled conditions, it is not sufficient to measure only its overall diagnostic accuracy. Equally important is understanding *how* and *under what circumstances* the system arrives at its diagnostic conclusions. Given that the checker interacts iteratively with users through a sequence of symptom-related questions, its diagnostic outcome depends both on the completeness of the provided information and on the structure of the underlying disease–symptom relations.

Therefore, this study investigates three complementary questions:

- **Convergence probability (Q1)** What is the probability that the symptom checker converges on the correct diagnosis when given the correct symptoms with a certain probability?
- **Convergence speed (Q2)** How quickly does it converge?
- **Understanding convergence probability and speed (Q3)** What features of the disease–symptom mapping affect the convergence probability and convergence speed?

Together, these questions aim to characterize both the robustness and efficiency of the inference process and to identify structural factors that may limit or enhance the system’s diagnostic performance.

The remainder of this paper is organized as follows. Section “Materials and methods” outlines the knowledge database and its disease–symptom mappings, describes the knowledge-based inference algorithm used by the symptom checker, and introduces the overall validation approach and its conceptual framework. Section “Results” presents the experimental setup and the quantitative results addressing the three research questions. Section “Discussion” discusses the implications of the findings, methodological limitations, and potential improvements of the system. Section “Related work” discusses the results in the context of related work. Finally, Section “Conclusions” concludes the paper and outlines directions for future work.

## Materials and methods

### The knowledge database

The symptom checker algorithm is based on relational knowledge database provided by veterinary experts. It implements the following relations:

$$\begin{aligned} \textit{disease2symptoms}&: D \rightarrow 2^{S} \\ \textit{symptom2diseases}&: S \rightarrow 2^{D} \\ \textit{location}&: D \rightarrow 2^{L} \\ L&= \big \{\text {``General / Whole body''},\, \text {``Skin / Fur''},\\&\qquad \text {``Head / Neck''},\, \text {``Back / Tail''},\, \text {``Chest''},\\&\qquad \text {``Abdomen / Pelvis''},\, \text {``Legs''}\big \} \\ \textit{confidence}\_\textit{disease}&: D \rightarrow \{\textit{low},\, \textit{medium},\, \textit{high}\} \\ \textit{confidence}\_\textit{symptom}&: S \times D \rightarrow \{\textit{low},\, \textit{medium},\, \textit{high}\} \end{aligned}$$

*D* is a set of diseases and *S* a set of symptoms, $2^{X}$ denotes the power set of *X*, i.e., the set of all subsets of *X*. The mapping *disease2symptoms* assigns to each disease the subset of symptoms that are typically observed for that disease. Conversely, *symptom2diseases* associates each symptom with the set of diseases in which it may occur. The *location* maps each disease to one or several anatomical regions. The mapping *confidence_disease* categorizes diseases according to their estimated prevalence in the population ($\textit{low}=0.25$, $\textit{medium}=0.5$, or $\textit{high}=0.75$). Finally, *confidence_symptom* expresses, for each symptom–disease pair (*s*, *d*), the confidence that symptom *s* is observed in disease *d*. Note that the confidence values of diseases and symptoms are unnormalized scores used for ranking and should not be interpreted as probabilities.

**Fig. 1:**
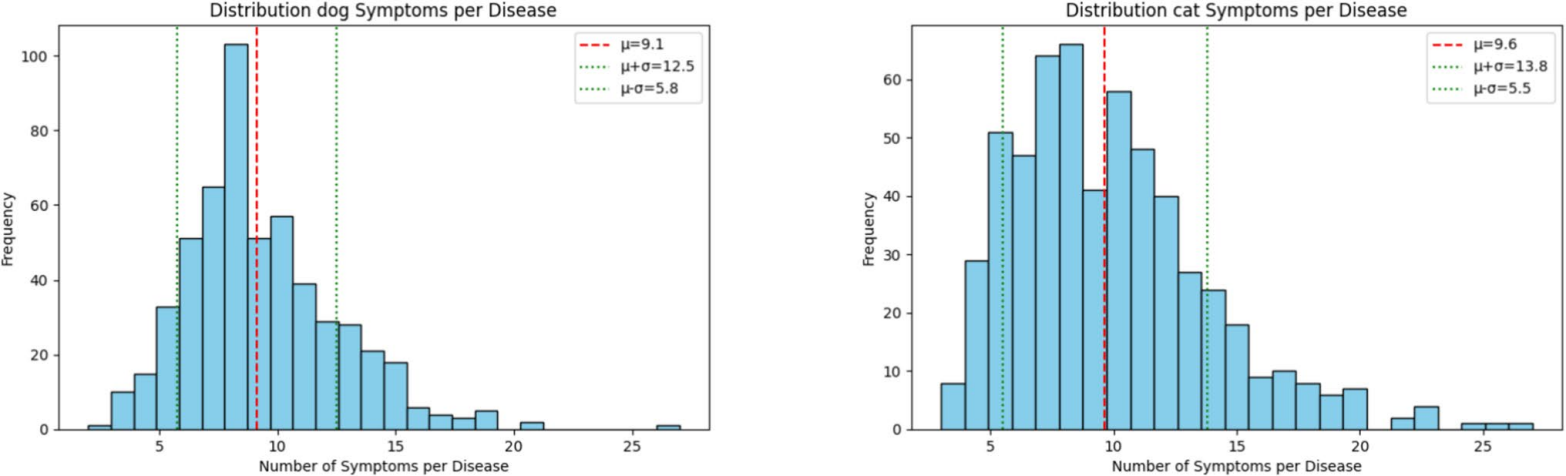
Symptom distributions per disease in dogs (left) and cats (right). The red dashed line shows the mean ($\mu$), and green dotted lines show $\mu \pm \sigma$

Note that there are different diseases and symptoms in dogs and cats, although they overlap to a large degree. Statistics about the diseases and symptoms in dogs and cats are summarized in Table 1.^1^ The distributions of the number of symptoms for each disease in dogs and cats are shown in Fig. 1. All data can be explored on https://petsvetcheck.de/en.

**Table 1:** Disease and symptom statistics

Metric	Dogs	Cats
Total # diseases	542	570
Total # unique symptoms	1062	1062
Diseases per location:		
General / Whole body	145	195
Skin / Fur	46	54
Head / Neck	133	122
Back / Tail	16	13
Chest	76	84
Abdomen / Pelvis	143	150
Legs	26	34
Diseases with one location	92.1%	86.7%
Symptoms per disease:		
Minimum	2	3
Maximum	27	27
Mean, standard deviation ($\mu \pm \sigma$)	$9.15\pm 3.36$	$9.64 \pm 4.15$

### The symptom checker algorithm

The symptom checker algorithm is an adaptive question-asking system that uses weighted updates to disease and symptom confidence values. It implements an iterative Bayesian-style confidence update procedure for symptom-based disease confidence estimation, given the answered questions after the symptoms observed. The algorithm starts with initial confidence values for diseases and symptoms set by experts. It repeatedly asks the user about the highest-confidence symptom. Each answer reshapes the confidence value distributions of diseases and symptoms: if the symptom is observed, it increases the confidence values of diseases connected to that symptom. If the symptom isn’t observed, it decreases them. No updates if the user isn’t sure. Anyway, the algorithm removes the asked symptom and updates the confidence values of the remaining symptoms.

Over time, the algorithm narrows down to the highest-confidence disease(s). Therefore, it proceeds in rounds where in each round, the highest-confidence (not necessarily the most informative) symptom is queried, and the disease and symptom confidence values are updated based on the answer. The main symptom checker loop works as follows:

1. **Select next symptom** At each iteration, the symptom with the highest current confidence value is selected.
2. **Check for stopping** If the confidence value of this symptom is too low, the process terminates.
3. **Obtain answer** An answer is requested from the user; in the validation, the user’s behavior is simulated with an automated answering strategy. The different strategies are defined in Section “The evaluation method”, but in the real-world system, this would be user input. The answer is “yes”/“no”/“don’t know” to the symptom being present.
4. **Update confidence values** of symptoms and diseases as follows. For diseases *d*: Each disease confidence value *C*(*d*) is updated depending on whether the answer was “yes” or “no” and on the confidence of the symptom occurring for the disease. The confidence value remains unchanged if the answer was “don’t know”. The *k* highest-confidence diseases are presented to the user:
  $$ C'(d) = {\left\{ \begin{array}{ll} C(d) \cdot f & \text {if answer = yes}, \\ C(d) \cdot (1-f) & \text {if answer = no}, \\ C(d) & \text {if answer = don't know}. \end{array}\right. } $$
  $$ f \in \{0.75, 0.5, 0.25\} $$
  For symptoms *s*: The asked symptom is removed from the candidate pool. For each remaining symptom, its confidence *C*(*s*) is updated according to the updated confidences $C'(d)$ of the associated diseases *d*. This means that symptoms linked to high-confidence diseases also become more likely to be asked next.

The rationale for choosing the above approach is to keep the user engaged and willing to provide additional information, as the system queries symptoms that are likely to be present. Alternatives could instead evaluate the prevalence of the most informative symptoms using Bayesian experimental design (Chaloner and Verdinelli 1995): the probability of each disease, computed via Bayesian inference, is interpreted as the current state of information, and the objective is to maximize the expected information gain by querying a particular symptom or even a set of symptoms simultaneously, as suggested by Guan and Baral (2021). While these and related approaches appear promising for improving performance, the present work focuses on evaluating the proposed lightweight symptom-checker algorithm regarding its convergence behavior and the relative ranking of suggested and actual diseases. This way, it establishes a validation methodology before leveraging it to benchmark alternative symptom-checking approaches in future work.

### The evaluation method

All analyses are performed separately for dogs and cats to account for species-specific differences.

Research questions **Q1** and **Q2** are addressed by designing a number of suitable assessment metrics, cf. Section “Assessment metrics”, defining a user agent with different answering strategies, cf. Section “Agent behavior”, simulating the dialog between the user agents and the symptom checker, assessing the metrics, and evaluating them both statistically and visually, cf. Sections “Results” and “Discussion”. Here, we also assess the systems runtime.

Research question **Q3** is addressed using classic hypothesis testing, applying regression analysis, Pearson’s correlation test, and ANOVA.

#### The statistical approaches

For each dog or cat disease, we run a dialog between the chosen user agent and the symptom checker algorithm introduced in Section “The symptom checker algorithm”. Each such run represents an independent execution of the algorithm.

We use regression analysis and Pearson’s correlation test and Kendall’s $\tau$ for assessing the impact of the numerical variables, e.g., the number of symptoms of a disease and the uniqueness of this symptom vector, on the convergence probability and speed.

We use ANOVA for assessing the impact of disease location on the convergence probability and speed, more specifically, the Python SciPy Statistical Function f_oneway which performs a one-way ANOVA test. The one-way ANOVA tests the null hypothesis that two or more groups have the same population mean. The test is here applied to samples from more than two groups (diseases at different locations) with differing sizes.

The parametric ANOVA tests assumes (i) independence of observations: each sample comes from an independent subject or trial, (ii) normality: the dependent variable (within each group) should be approximately normally distributed, and (iii) homogeneity of variances: the variance of the dependent variable should be similar across all groups.

However, 7.9% of the dog diseases and 13.3% of the cat diseases affect multiple locations, cf. Table 1, violating (i) and precluding naive use of ANOVA tests across all diseases. One approach is to restrict the analysis to single-location diseases. While this approach preserves (i), it includes only 92.1% and 86.7% of the diseases for dogs and cats, resp. Therefore, we incorporate multi-location diseases by defining combined location categories, provided each combination includes a sufficient number (we chose $>10$) of diseases to ensure stable rank distributions; rarer combinations were collapsed into an “Other multi-location” group to avoid small-sample artifacts. The “Other multi-location” groups contains 31 (5.7% of the) diseases for dogs and 43 (7.5% of the) diseases for cats, resp. This strategy allows meaningful ANOVA inference, while respecting the full set and the structure of the data and maintaining interpretability, enabling assessment of how body location relates to convergence probability and speed.

While all (ii) and (iii) could be tested with further statistics, we simply opted to also apply the non-parametric Kruskal-Wallis test, also relying on (i) but not requiring (ii) and (iii) to achieve the necessary robustness.

Kruskal-Wallis tests the null hypothesis that the population median of all groups are equal. It is a non-parametric version of ANOVA. This test also works on more than two independent samples with different sizes. For this non-parametric ANOVA test, we use the Python SciPy function kruskal.

Rejecting the null hypothesis does not indicate which of the groups (disease locations) differs. Post hoc comparisons between groups are required to determine which groups are different. Therefore, we used the Python Pingouin function pairwise_tests to perform pairwise Mann–Whitney U tests between any pair of locations. Each pairwise comparison produces a *p*-value, i.e., the probability of observing the data if there were actually no difference between the groups.

However, when conducting many such tests simultaneously, the probability of obtaining at least one false positive increases simply by chance. To control this inflation of error, we apply Bonferroni’s *p*-value correction method, controlling the family-wise error rate (probability of any false positive). We report both the uncorrected $p_\textrm{unc}$ and the corrected $p_\textrm{cor}$ values, but in our conclusions, we only rely on the corrected ones. Note that under Bonferroni correction, each *p*-value is multiplied by the number of pairwise comparisons. As a result, moderately large uncorrected *p*-values can exceed 1 after correction and are therefore truncated to 1.00.

Finally, to identify the most predictive structural properties (features) of the diseases impacting their diagnosis convergence probability and speed, we trained a Random Forest regression model in a cross-validated setting. All $P=4$ properties (disease frequency, location, number of symptoms, and uniqueness of the related symptom vectors) were used as predictors for the target variable measuring the difficulty of a disease to be identified. To this end, we design a corresponding response score capturing the “disease difficulty”, cf. Section “Assessment metrics”.

A five-fold cross-validation was used (implemented in Python in the Sklearn function KFold) to assess the model’s stability and generalization. For each fold, the training subset was used to fit a Random Forest Regressor (Sklearn’s RandomForestRegressor) with the following configuration: 300 trees, a maximum tree depth of 6, a minimum of 10 samples per split and 5 samples per leaf, a maximum $\sqrt{P}=2$ of all $P=4$ structural properties each split in a tree, and each tree is trained on a random sample containing at most $80\%$ of the available training data to introduce additional bagging variability.

The predictive performance of the model was evaluated using the coefficient of determination ($R^2$) under 5-fold cross-validation. The mean $R^2$ represents the proportion of variance in the disease difficulty score that can be explained by the selected features across all folds; its standard deviation reflects the stability of this relationship across different folds.

Feature importance values were extracted from each fitted model. Feature importance was quantified using the built-in impurity-based measure of the Random Forest model, which reflects the average reduction in prediction error (mean squared error) attributed to splits on each feature across all trees. To ensure robustness, feature importance was averaged across five cross-validation folds. This yielded a ranked list of predictors, where higher importance values indicate a stronger contribution to explaining the variance in the disease difficulty score.

In addition, Pearson’s correlation coefficient (*r*) was computed between each feature and the target variable to provide an independent measure of linear association.

The resulting feature importance reports the mean and standard deviation of each feature’s contribution to the model, along with its correlation with the target variable. This allows interpretation of both the model-based and statistical relevance of each predictor in explaining variance in disease scores.

For all tests, we employ visual analytics, an interactive and iterative process in which data and assessment results are visualized to gain insights, possibly creating new questions and hypotheses that, in turn, lead to new tests and visualizations.

#### Assessment metrics

To answer **Q1** and **Q2**, the performance of the symptom checker was evaluated using the following metrics:

- **Rank10:** The rank position of the correct diagnosis/disease after 10 questions. A lower rank indicates that the correct disease is prioritized early in the diagnostic process.
- **Rank25:** The rank position of the correct diagnosis/disease after 25 questions. This metric evaluates how the symptom checker refines its predictions as more information is obtained.
- **Convergence:** The number of questions required for the symptom checker to converge on the correct diagnosis/disease *d*. Convergence is defined conservatively as the point at which *d* achieves the highest confidence score *C*(*d*) with good margins, i.e., for the disease $d'$ with the second highest confidence score $C(d')$, it holds $C(d')< 0.25 C(d)$.
- **Convergence Ratio:** The proportion of diseases for which the symptom checker successfully converges. This provides an overall measure of the system’s reliability across all diseases.
- **Top3:** The number of questions needed until the correct disease is identified among the three diseases with the highest confidence score reflects how quickly the correct diagnosis enters the symptom checker’s shortlist of highest-confidence candidates.

Each disease is evaluated using the four performance metrics: Rank10, Rank25, Convergence, and Top3. Recall that the Convergence Ratio is a system metric, not one for any individual disease.

While the individual metrics capture different aspects of diagnostic behavior, they may correlate, i.e., overlap in the information they convey, leading to redundancy and difficulties in direct comparison across diseases. Therefore, to obtain an overall performance measure for each disease, a composite disease difficulty score was derived, allowing us to address **Q3**.

To obtain this overall performance score for each disease, a composite score was derived using Principal Component Analysis (PCA)^2^ applied to the individual evaluation metrics. Let the matrix $\textbf{M} \in \mathbb {R}^{n \times 4}$ represent the metrics for *n* diseases, where each row corresponds to a disease and the columns correspond to

$$ [{\textbf {Rank10}}, {\textbf {Rank25}}, {\textbf {Convergence}}, {\textbf {Top3}}]. $$

Each column was standardized to zero mean and unit variance prior to PCA. The first principal component, capturing the largest proportion of variance across metrics, was used as a composite performance measure.

For each disease $d_i$, the composite score was computed as $\text {score}(d_i) = \textbf{w}^\top \textbf{m}_i$, where $\textbf{w}$ denotes the loading vector of the first principal component and $\textbf{m}_i$ is the standardized metric vector for disease $d_i$. The resulting overall disease difficulty score:

$$ {\textbf {disease}}\_{\textbf {scores}} = \{\, d_i \mapsto \text {score}(d_i) \,\} $$

provides a single PCA-based composite score per disease, summarizing its relative diagnostic performance across all evaluation metrics, with higher values indicating the difficulty of diagnosis.

In this study, the first principal component accounted for a substantial majority of the total variance in the four (standardized) evaluation metrics, 78.2% for dogs and 73.23% for cats, indicating a strong shared structure across the individual evaluation metrics. This justifies the use of the first principal component as a single composite score, as it captures the dominant pattern of variation while avoiding overemphasis on any individual metric. Consequently, ${\textbf {disease}}\_{\textbf {scores}}$ provides a robust and interpretable aggregation of the difficulty in diagnosing a disease.

Finally, also for understanding the reasons for (difficult) convergence, a lack of uniqueness score was derived. It quantifies how similar a disease is to all other diseases based on their symptom vectors. For each pair of diseases, a similarity score is computed (using cosine similarity), and the total similarity of each disease to all others is aggregated. This high ${\textbf {lack}}\_{\textbf {of}}\_{\textbf {uniqueness}}$ value indicates that a disease shares many symptoms with other diseases and is, thus, (hypothetically) harder to distinguish, whereas a low value reflects a more distinctive symptom profile. Formally, the score for a disease *d* is given by the sum of its pairwise similarity values with all other diseases. The resulting metric:

$$ {\textbf {lack}}\_{\textbf {of}}\_{\textbf {uniqueness}} = \{\, d_i \mapsto \sum _{j\ne i} \text {similarity}(d_i, d_{j}) \,\} $$

#### Agent behavior

The symptom checker user agent can simulate different answering behaviors using the following strategies:

- **CORRECT:** Always answers correctly. If the asked symptom *s* is present in the set of symptoms $\textit{disease2symptoms}(d)$ of the true disease *d*, the answer is “yes”; otherwise, “no”. This strategy represents an ideal responder with perfect knowledge.
- **HIGH:** Answers “yes” iff the symptom $s \in \textit{disease2symptoms}(d)$ for the correct disease *d* and its $\textit{confidence}\_\textit{symptom}(s,d)\in \{\textit{high}\}$. Otherwise, it answers “no”. It strategy simulates a conservative responder who only acknowledges highly probable symptoms.
- **HIGH_MEDIUM:** Answers “yes” iff the symptom $s \in \textit{disease2symptoms}(d)$ for the correct disease *d* and its $\textit{confidence}\_\textit{symptom}(s,d)\in \{\textit{high}, \textit{medium}\}$, otherwise “no”. Compared to HIGH, this strategy is less conservative and accepts symptoms with moderate confidence.
- **PROB_CORR:** Answers probabilistically based on $\textit{confidence}\_\textit{symptom}(s,d)$ for the asked symptom *s* and the correct disease *d*: if $s \in \textit{disease2symptoms}(d)$, it answers “yes” with probability equal to $\textit{confidence}\_\textit{symptom}(s,d)$; otherwise, it answers “don’t know”. It always answers “no” if $s \not \in \textit{disease2symptoms}(d)$. This strategy models an uncertain but generally correct responder.
- **PROB:** Answers probabilistically without allowing a “don’t know” response: if $s \in \textit{disease2symptoms}(d, s)$, it answers “yes” with probability equal to $\textit{confidence}\_\textit{symptom}(s,d)$. Otherwise, it answers randomly “yes” or “no” each with probability 0.5. This models a responder whose answers reflect uncertainty but are forced to choose “yes” or “no”.

Table 2 summarizes the different agent strategies assessed.

**Table 2:** Probability of “yes” and “don’t know” answers for a symptom *s* and a connected disease *d* in the different agent answering strategies **given** $s \in \textit{disease2symptoms}(d)$

Strategy	Prob. “yes”	Prob. “don’t know”
CORRECT	1	0
HIGH	$${\left\{ \begin{array}{ll} 1, & \text {if } \textit{confidence}\_\textit{symptom}(s,d) \in \{\textit{high}\} \\ 0, & \text {otherwise} \end{array}\right. }$$	0
HIGH_MEDIUM	$${\left\{ \begin{array}{ll} 1, & \text {if } \textit{confidence}\_\textit{symptom}(s,d) \in \{\textit{high}, \textit{medium}\} \\ 0, & \text {otherwise} \end{array}\right. }$$	0
PROB_CORR	$\textit{confidence}\_\textit{symptom}(s,d)$	$1-\textit{confidence}\_\textit{symptom}(s,d)$
PROB	$(1+\textit{confidence}\_\textit{symptom}(s,d))/2$	0

## Results

### Assessing convergence probability and speed (Q1, Q2)

We run $5 \times 542=2710$ dialogs—each a full execution of the symptom checker algorithm, cf. Section “The symptom checker algorithm”—to test the 5 answering strategies in the 542 dog diseases and $5 \times 570=2850$ dialogs to test the same answering strategies in the 570 cat diseases.

All tests are run locally on a MacBook Pro with a 2.6 GHz 6-Core Intel Core i7 processor and 32 GB 2667 MHz DDR4 memory. One test run for all dog (cat) diseases and one agent strategy takes about 115.5 sec. (147.6 sec.), i.e., 0.213 msec. (0.258 msec.) per dog (cat) disease. While the runtime measurements were obtained under a single hardware and software configuration, the symptom checker’s response time is most likely not an issue.

All results discussed below reflect the idealized answering strategies. These results should not be interpreted as directly transferable to real-world usage, where user responses may be incomplete, uncertain, or inconsistent beyond what was reflected in these answering strategies.

For the converging cases, the assessment metrics corresponding to each answering strategy are summarized in Table 3 for dogs and in Table 4 for cats, respectively. For the non-converging cases, the mean ranks of the correct disease after algorithm termination and their standard deviations are summarized in Table 5.

**Table 3:** Performance metrics in dogs (converging cases)

Strategy	Converge	Rank10	Rank25	Convergence	Top3
	Ratio (%)	($\mu \pm \sigma$)	($\mu \pm \sigma$)	($\mu \pm \sigma$)	($\mu \pm \sigma$)
CORRECT	100.0	3.19 ±5.58	1.20 ±1.25	13.89 ±9.05	6.42 ±6.38
HIGH	94.7	5.17 ±8.88	2.03 ±3.96	18.49 ±14.32	8.68 ±10.78
HIGH_MEDIUM	99.8	3.24 ±5.75	1.20 ±1.25	13.83 ±9.11	6.42 ±6.46
PROB_CORR	97.6	4.74 ±7.74	1.60 ±2.08	22.08 ±16.48	9.02 ±9.60
PROB	90.4	7.23 ±12.98	3.68 ±9.47	22.58 ±21.26	11.15 ±18.12

**Table 4:** Performance metrics in cats (converging cases)

Strategy	Converge	Rank10	Rank25	Convergence	Top3
	Ratio (%)	($\mu \pm \sigma$)	($\mu \pm \sigma$)	($\mu \pm \sigma$)	($\mu \pm \sigma$)
CORRECT	100.0	2.58 ±4.09	1.08 ±0.50	13.70 ±9.04	5.74 ±5.61
HIGH	92.8	3.83 ±6.88	1.71 ±3.12	17.51 ±14.10	7.25 ±9.29
HIGH_MEDIUM	99.4	2.70 ±4.55	1.11 ±0.71	13.80 ±9.30	5.81 ±5.85
PROB_CORR	97.7	3.41 ±5.29	1.29 ±1.07	19.79 ±14.47	7.06 ±7.80
PROB	93.3	4.98 ±10.64	3.04 ±7.27	20.84 ±21.61	7.71 ±11.89

**Table 5:** Final ranks of correct diseases in Non-Converging Cases (#NC) for dogs and cats

Strategy	Dogs (all cases: 585 )		Cats (all cases: 652)	
	#NC	Rank ($\mu \pm \sigma$)	#NC	Rank ($\mu \pm \sigma$)
HIGH	30	2.43 ± 1.55	47	2.94 ± 4.41
HIGH_MEDIUM	1		4	1 ± 0
PROB_CORR	14	1.18 ± 0.40	20	1.10 ± 0.31
PROB	53	6.38 ± 10.53	39	4.56 ± 9.72

**Fig. 2:**
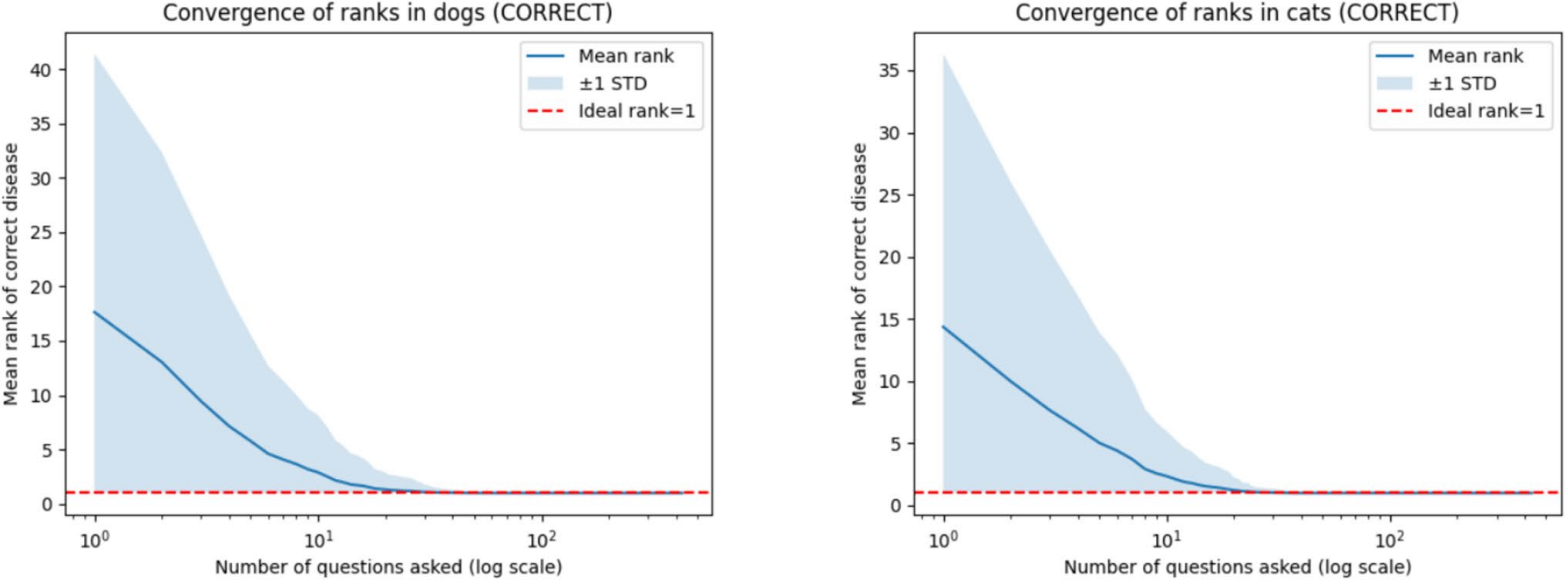
Distribution of disease ranks (mean and 1 standard deviation) over the number of symptoms asked (log scale) in dogs (left) and cats (right)

The performance metrics Rank10, Rank25, Convergence, and Top3 reveal different discrete aspects of convergence performance. To complement these with continuous assessment, Fig. 2 shows the distribution of disease ranks (mean and one standard deviation) over the number of symptoms asked in a dialog between the symptom checker and a (simulated) user in dogs and cats, respectively. Mind the log-scale on the x-axis.

For both dogs and cats, the ranks drop to the top rank on average after $\approx 20$ questions under the CORRECT answering strategy, i.e., in the idealized simulation setting with perfectly consistent answers. This reflects the theoretically optimal convergence behavior of the algorithm in this controlled simulation setting.

The other answering strategies show similar behavior, though with slightly slower convergence (omitted here). The 1-standard deviation intervals also decrease with the increasing number of questions, indicating that more diseases have converged to the top rank.

### Understanding convergence probability and speed (Q3)

**Fig. 3:**
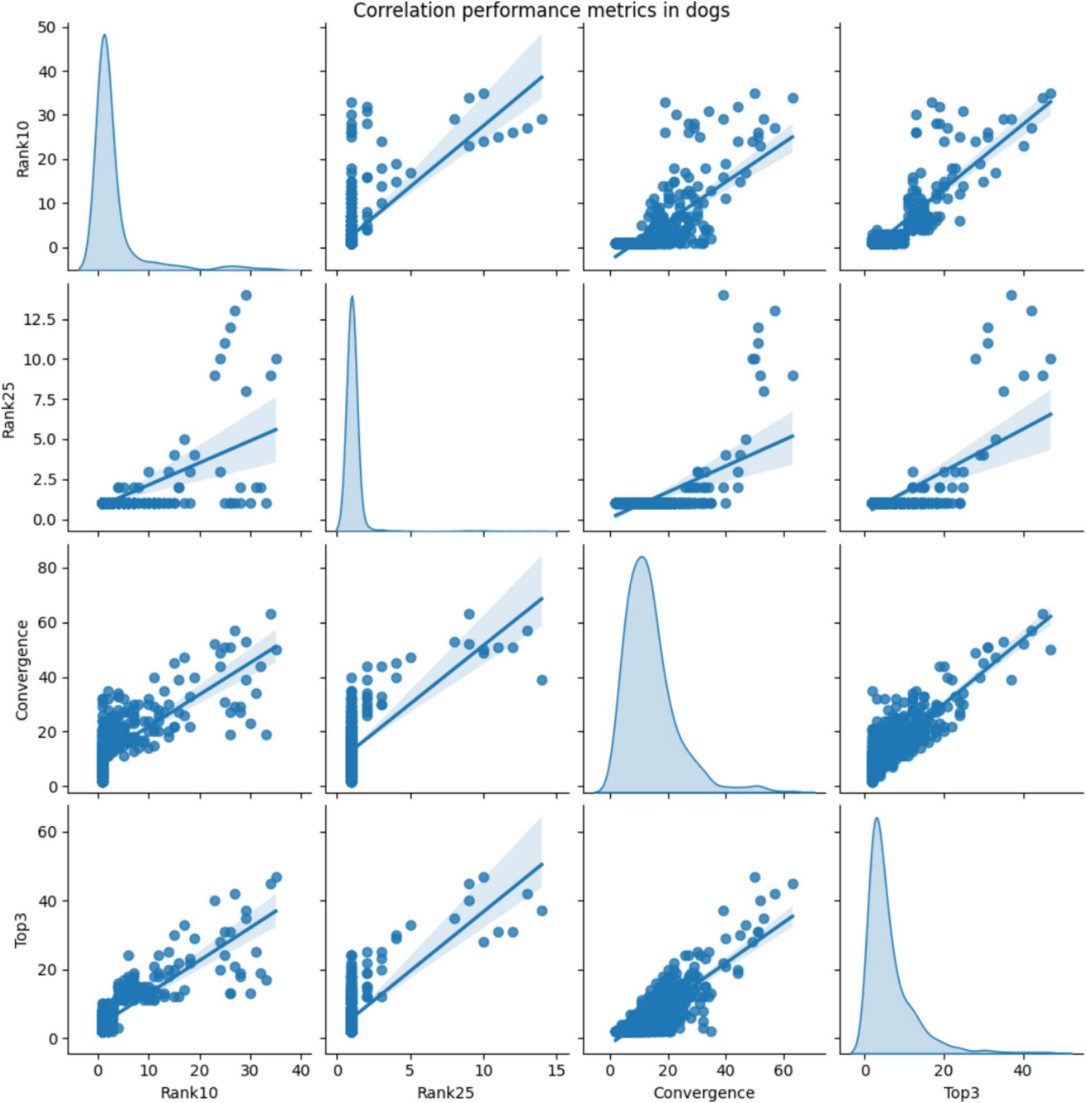
Distribution of assessment metrics (diagonal) and their pairwise correlation (off diagonal) in dogs for the CORRECT answering strategy

As expected, the performance metrics Rank10, Rank25, Convergence, and Top3 are highly correlated, as confirmed by the correlation diagrams (exemplified for dogs and answering strategy CORRECT) in Fig. 3. This justifies the PCA-based aggregation of these individual metrics to the common disease_score, cf. the definition in Section “Assessment metrics”, reflecting how difficult it is for a disease to float to the top ranks during user interaction with the symptom checker.

**Fig. 4:**
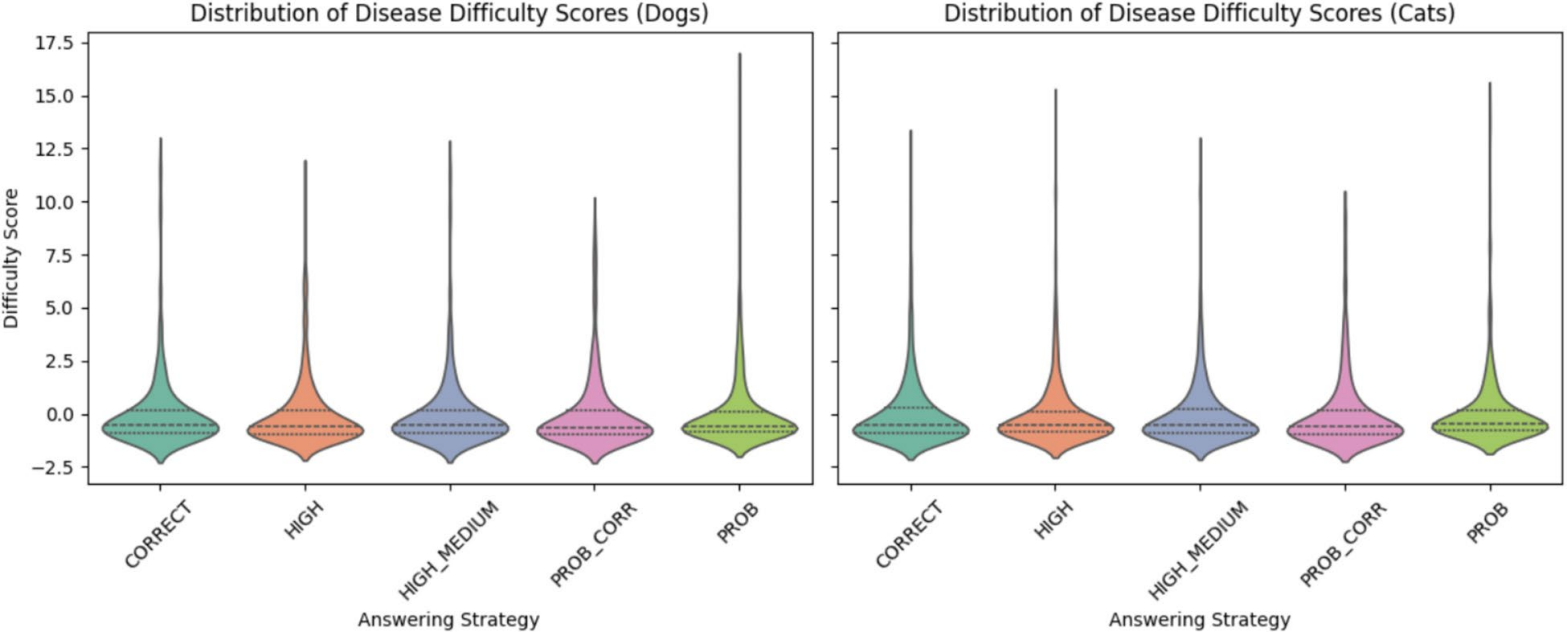
Distributions of disease scores for the different answering strategies in dogs (left) and cats (right)

With this aggregated score of the “difficulty” of diseases, we can assess the distribution of easy and difficult diseases in dogs and cats, respectively, for the different answering strategies. These distributions are displayed in Fig. 4 using violin plots.^3^ The *x*-axis shows the answering strategy, the *y*-axis represents the disease_score ranging from low (easy to identify) at the bottom to high (difficult to identify) at the top. Each violin shows how the disease difficulty scores are distributed for that strategy. We observe that the distributions are quite similar: all are skewed towards easier diseases, with a long tail of a few difficult ones.

Now, we are ready to approach the question of which features of the disease–symptom mapping affect disease difficulty, i.e., the convergence probability and speed. Understanding that the differences in the results for the different answering strategies are rather low, the subsequent analyses will only be displayed for the CORRECT strategy.

**Fig. 5:**
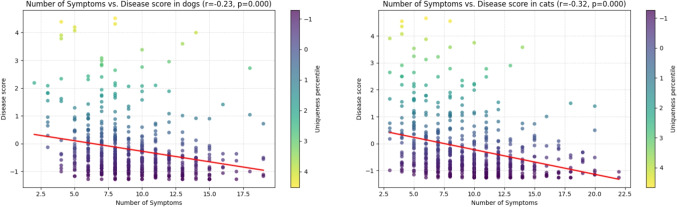
Number of symptoms per disease to disease_score in dogs (left) and cats (right)

First, we test the hypothesis that the number of symptoms is positively related to convergence, i.e., negatively to the disease score. Figure 5 shows the linear dependence between these two variables and the results of correlation analysis.

Assuming low correlation is indicated by $$|r| \approx 0.1$$–0.3 and medium correlation by $$|r| \approx 0.3$$–0.5, we observe a low negative correlation in dogs ($$r=-0.23)$$ and a low to medium negative correlation in cats ($$r=-0.32)$$ both with high significance ($$p<0.0005)$$.

Assuming low correlation is indicated by $|r| \approx 0.1$–0.3 and medium correlation by $|r| \approx 0.3$–0.5, we observe a low negative linear correlation in dogs ($r=-0.23$, $p<0.0005$) and a low-to-medium negative linear correlation in cats ($r=-0.32$, $p<0.0005$).

Complementary rank-based analyses using Kendall’s $\tau$ reveal a weak negative monotonic association between the number of symptoms and disease score in dogs ($\tau =-0.191$, $p<0.0001$) and a weak-to-moderate negative monotonic association in cats ($\tau =-0.256$, $p<0.0001$), following common interpretations for Kendall’s $\tau$, where $|\tau | \approx 0.10--0.20$ indicates a weak association and $|\tau | \approx 0.20-0.35$ a moderate association.

The negative signs of *r* and $\tau$ indicate that disease score tends to decrease as the number of symptoms increases, while the comparison between low Pearson *r* and Kendall’s $\tau$ suggests that the relationships are monotonic but not strictly linear.

**Fig. 6:**
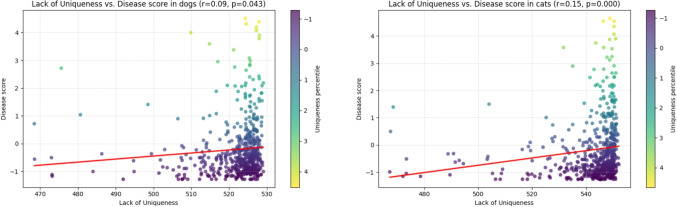
Lack of disease uniqueness to disease_score in dogs (left) and cats (right)

Next, we test the hypothesis that the uniqueness of the symptoms mapped to a disease is positively related to convergence, i.e., the lack of uniqueness is positively correlated to the disease (convergence difficulty) score. To this end, we use the diseases’ lack of uniqueness score defined in Section “The evaluation method” as the independent variable. Figure 6 shows the linear dependence between these two variables using linear regression and the results of the correlation analysis.

Assuming negligible correlation is indicated by $|r| < 0.1$, we observe a negligible to low positive correlation in dogs ($r=0.09$) with a significance level of $p<0.05$ and a low positive correlation in cats ($r=0.15$) with high significance ($p<0.0005$). Complementary rank-based analyses using Kendall’s $\tau$ reveal weak positive monotonic associations between lack of uniqueness and disease score in both dogs ($\tau =0.130$, $p<0.0001$) and cats ($\tau =0.191$, $p<0.0001$). The positive signs of *r* and $\tau$ indicate that disease score tends to increase as uniqueness decreases, while the comparison between low Pearson *r* and Kendall’s $\tau$ suggests that the relationships are monotonic but not strictly linear.

We also note a skewed distribution of diseases towards those lacking uniqueness.

**Fig. 7:**
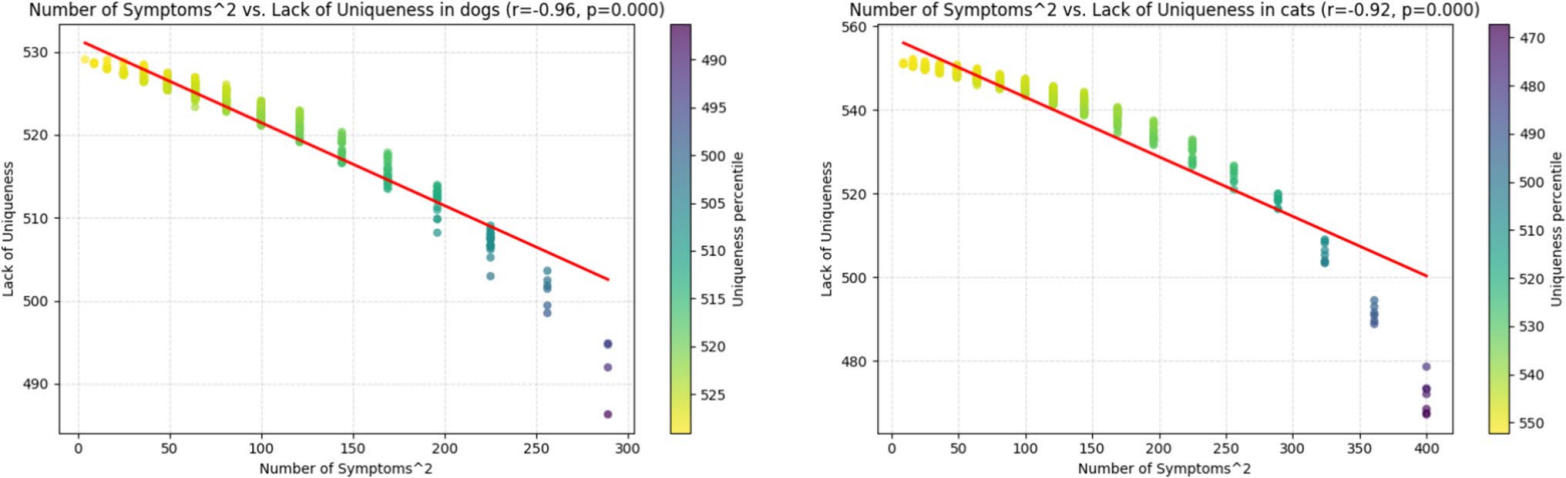
Squared number of symptoms per disease to lack of disease uniqueness in dogs (left) and cats (right)

The similarity of the results concerning the dependence of the disease difficulty score on the number of symptoms and on symptom uniqueness, respectively, is not unexpected. Intuitively, diseases characterized by a large number of symptoms are less likely to share identical symptom patterns with other diagnoses than diseases defined by only a few symptoms. This assumption is strongly supported by the observed association between the (squared) number of symptoms and the lack of uniqueness. In dogs, this relationship is characterized by a very high negative linear correlation (Pearson’s $r=-0.958$, $p<0.0001$) and a correspondingly strong negative monotonic association (Kendall’s $\tau =-0.876$, $p<0.0001$). Similarly, in cats, we observe a very high negative linear correlation (Pearson’s $r=-0.922$, $p<0.0001$) together with a strong negative monotonic relationship (Kendall’s $\tau =-0.880$, $p<0.0001$). These results confirm that increasing symptom counts are tightly linked to increased diagnostic distinctiveness across both species. See also Fig. 7.

**Fig. 8:**
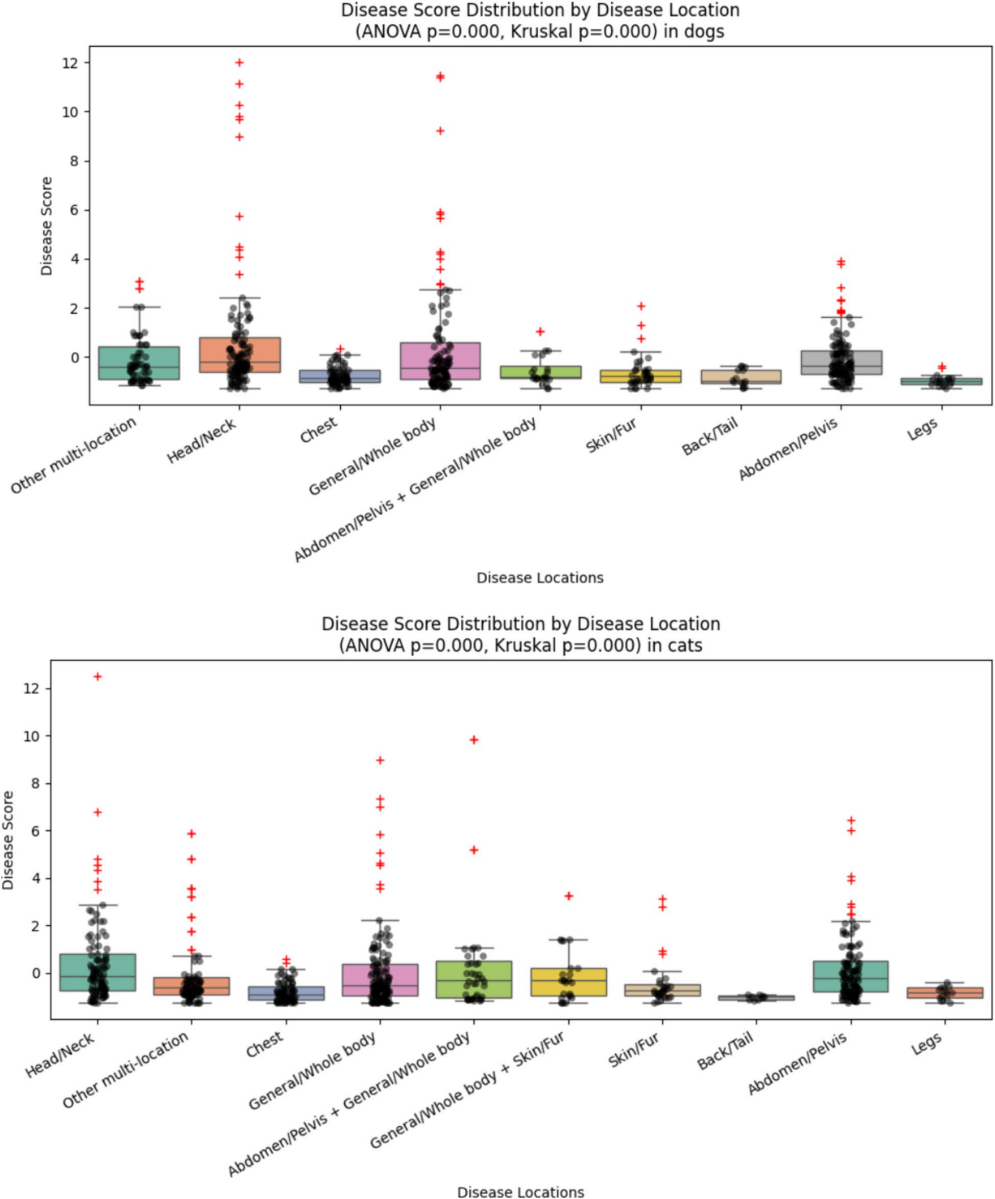
ANOVA in dogs (top) and cats (bottom)

Next, we test the hypothesis that the primary location of a disease is connected to the disease score. As “location” is a categorical feature, we use ANOVA for testing this hypothesis. Figure 8 displays the test results with boxplots: the boxes show the quintiles of the disease score distributions, the gray dots the individual disease score values; the red pluses mark outliers.

Both ANOVA tests, the parametric ANOVA ($p = 6.21\times 10^{-9}$) and the non-parametric Kruskal–Wallis ($p = 2.21\times 10^{-15}$), show high significance in dogs. This continues to hold for the cats’ parametric ANOVA ($p=8.36\times 10^{-06}$) and Kruskal–Wallis ($p=6.11\times 10^{-12})$. This lets us conclude that there are disease score differences between (at least two of) the disease locations, which was already evident when inspecting Fig. 8. Further analyses show pairwise differences in disease scores depending on the disease location for both species. Overall, the Chest and Head/Neck areas act as the primary drivers of significance, showing the most consistent differences against other body regions. Table 6 summarize the regions with significant differences.

**Table 6:**
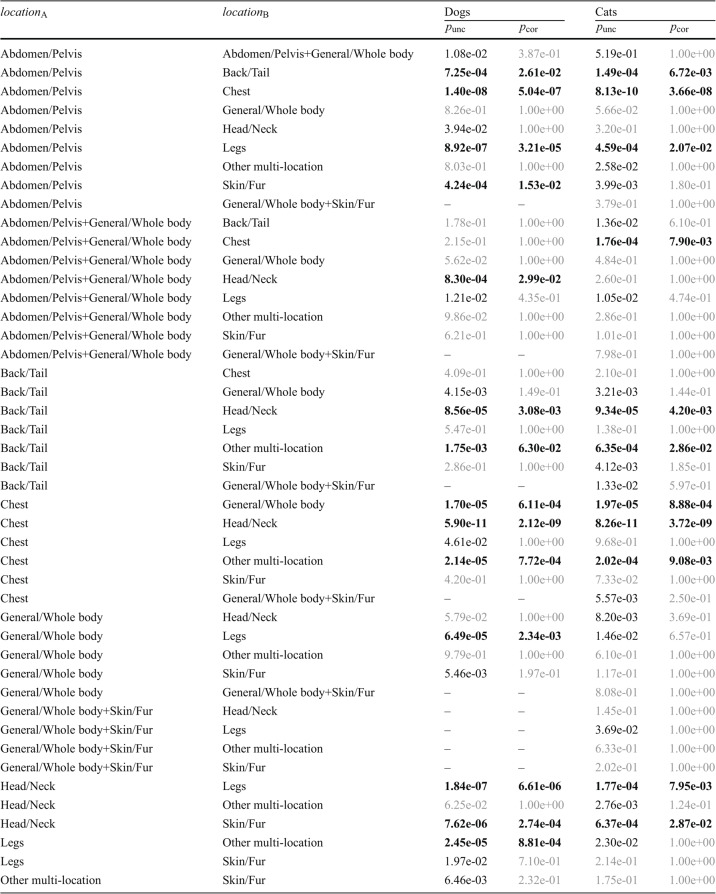
Pairwise comparisons of disease locations in dogs and cats. Boldface values are significant after Bonferroni correction ($p_\textrm{cor} < 0.05$); regular values are significant before correction ($p_\textrm{unc} < 0.05$ but $p_\textrm{cor} > 0.05$); gray values are not significant

We note that many corrected *p*-values reach 1, cf. the explanation of the correction in Section “The statistical approaches”. This can be explained by the number of pairwise comparisons. For dogs, we have $k=9$ (multi-) locations, for cats even $k=10$. The $k(k-1)/2$ pairwise comparisons lead to a multiplier of 36 for dogs and 45 for cats. Consequently, even moderately large uncorrected *p*-values can exceed 1 after correction (and are truncated to 1).

**Fig. 9:**
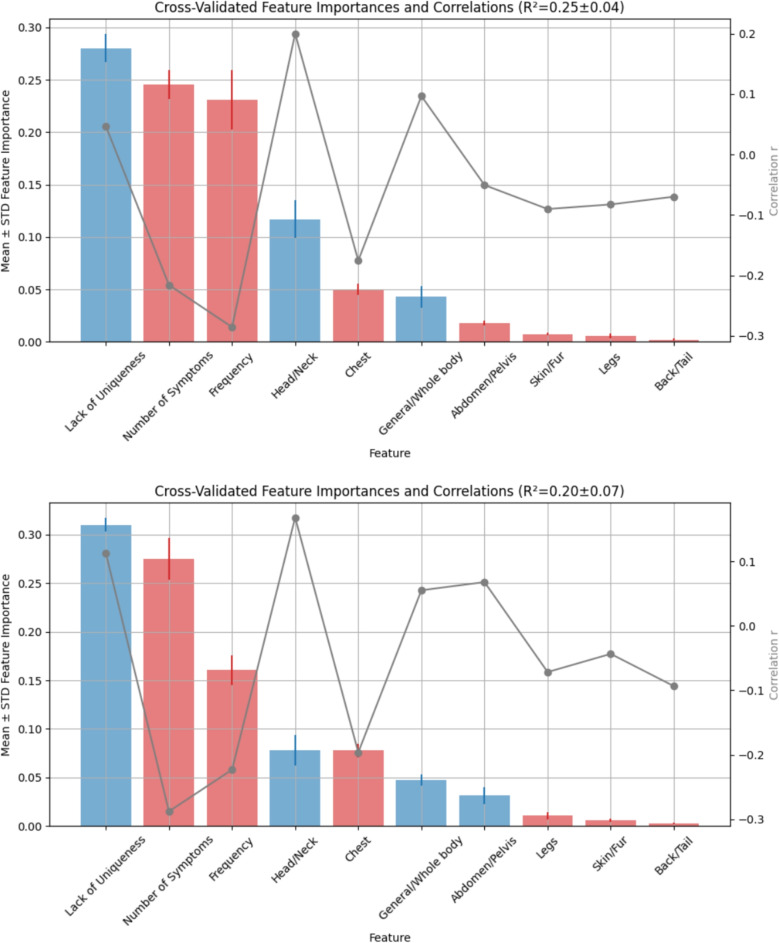
Feature importance and correlation with disease difficulty score in dogs (top) and cats (bottom). Bars show mean feature importance across five cross-validation folds with confidence intervals, colored by correlation direction (blue: positive, red: negative). Gray dots and lines indicate the Pearson correlation of each feature with the disease score. The $R^2$ in the title summarizes overall model performance

Finally, we analyze the feature importance of all variables analyzed so far individually for predicting the disease difficulty score, including the number of symptoms mapped from each disease, the uniqueness of these mapped symptom vectors, the disease frequency ($\textit{confidence}\_\textit{disease}$), and the disease location. To this end, we employ the Feature Importance analysis outlined in the methods Section “The evaluation method”. Figure 9 visualizes the results of this analysis.

The results show a weak relationship between the variables and the disease score in both dogs ($R^2=0.25\pm 0.04$) and cats ($R^2=0.2\pm 0.07$). Hence, the models would be of limited predictive value. However, the intention of our analysis is interpretation rather than prediction.

The feature importance ranking is almost the same for dogs and cats, with only the importance of the locations “Legs” and “Skin/Fur” being swapped. We note that the most important features are those already analyzed in detail: the number of symptoms (cf. Fig. 5) and the uniqueness of the symptom vectors (cf. Fig. 6).

Also, the importance of the features across the five cross-validation folds within dogs and cats, respectively, is quite stable, as the tight confidence intervals (lines on top of each feature bar) indicate.

The correlation between the individual features and the disease score varies among the features, as the gray dots indicate (connected with gray lines for better visibility). We already analyzed these correlations for the number of symptoms (cf. Fig. 5) and the uniqueness of the symptoms (cf. Fig. 6). The direction of the correlations of a feature with the disease score is visualized with the color of the corresponding bar: blue for positive correlations and red for negative correlations. This direction is almost the same for dogs and cats, with the “Abdomen/Pelvis” location as the only exception.

## Discussion

This section discusses the implications of the experimental findings, methodological limitations, and potential directions for system improvements.

### Discussing convergence probability and speed (Q1 & Q2)

The quantitative results presented in Section “Assessing convergence probability and speed (Q1, Q2)”, Tables 3-5, and Fig. 2 demonstrate that the system exhibits a high degree of robustness and computational efficiency. The convergence ratios achieved under near-ideal simulated user conditions were exceptionally high (for user agent behavior CORRECT: 100.0%; HIGH MEDIUM: 99.8% for dogs and 99.4% for cats), indicating strong internal consistency and correctness of the underlying inference rules when symptom inputs are reliable.

The rapid convergence behavior, where the mean disease rank reached one after approximately twenty questions under the CORRECT strategy, combined with very short response times ($0.213-0.258$ ms per disease), provides evidence for the system’s efficiency and practical suitability for initial triage scenarios.

Although we observed reduced performance for the probabilistic answering strategies (PROB CORR and PROB), the sensitivity of the system to uncertainty in user inputs is rather low.

Non-converging cases were rare, and the correct disease often remained among the top-ranked suggestions (typically ranks 1–6 for dogs and 1-4 for cats). This indicates that, even when convergence criteria were not met, the diagnostic ranking still provided clinically useful guidance.

Furthermore, the observed skew in disease difficulty scores towards generally “easy” diseases, with a long tail of harder ones, highlights the presence of a small subset of diseases that challenge the inference engine. This distribution suggests that targeted refinement of the knowledge base could improve performance on these outlier cases.

However, the lower convergence ratios and slower convergence speeds for the probabilistic answering strategies suggest that user uncertainty or symptom-reporting errors can affect reliability in real-world deployments. Clinical tests are therefore required and planned as future work.

### Discussing factors affecting convergence (Q3)

The structural analysis in Section “Understanding convergence probability and speed (Q3)” identified several factors influencing disease difficulty. A weak but consistent negative correlation between the number of symptoms per disease and the disease score ($r = -0.23$ for dogs, $r = -0.32$ for cats) implies that diseases described by more symptoms are generally easier to identify.

Conversely, the weak positive correlation between lack of uniqueness and disease score ($r = 0.09$ for dogs, $r = 0.15$ for cats) suggests that diseases sharing many symptoms with others are (slightly) harder to distinguish. This finding emphasizes the importance of symptom distinctiveness in the construction of the knowledge base.

The ANOVA results revealed highly significant differences in disease difficulty across anatomical regions, with several strong pairwise contrasts (e.g., Abdomen/Pelvis vs. Back/Tail, Chest, and Legs, resp.). This indicates that the diagnostic complexity varies by location, reflecting underlying structural or symptom-overlap differences in the data.

Feature importance analysis confirmed that the number of symptoms and their uniqueness were the most influential predictors of disease difficulty. The stability of these results across cross-validation folds and across both species (dogs and cats) supports their structural relevance to the inference process.

### Methodological limitations

Several limitations should be considered when interpreting these findings. First, the Random Forest regression model used to predict disease difficulty showed only limited explanatory power ($R^2 = 0.25 \pm 0.04$ for dogs and $R^2 = 0.20 \pm 0.07$ for cats). Although the models provided interpretable feature importance estimates, their predictive accuracy remains weak, suggesting that additional or more complex predictors may be needed to better explain diagnostic difficulty. This does not come as a surprise, as any results from paraclinical diagnostics including laboratory and imaging diagnostics are intentionally not included, since the symptom checker knowledge base was designed for non-experts (pet users, first-line support etc.).

Second, the validation relied on automated answering strategies. This approach guarantees repeatability and avoids issues related to limited availability of real-world cases, yet it introduces the limitation that the system’s performance has not been confirmed under authentic clinical or user conditions. Future validation will therefore include empirical data from veterinary practice and controlled user studies.

### Potential improvements

**Fig. 10:**
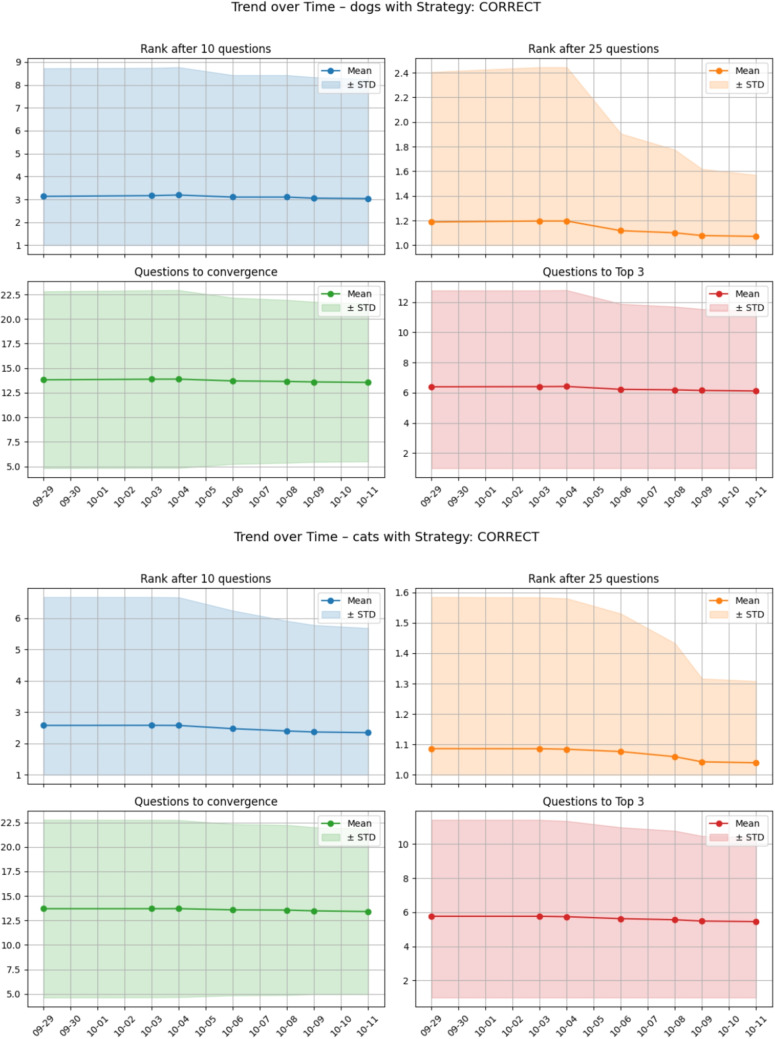
Improvements in dogs (top) and cats (bottom) for the CORRECT answering strategy after Oct. 4th, 2025 following the insights from the analysis of Q3

The results provide several concrete directions for improving the system. The identification of a small group of diagnostically difficult diseases suggests that refinement efforts should focus on these outliers—particularly those with low symptom uniqueness or inconsistent profiles. Expert review could ensure that these diseases are represented by more distinctive and discriminative symptom sets. This approach has actually already been taken to refine the knowledge database after the data from this analysis (Oct. 4th, 2025), leading to the improvements documented in Fig. 10.

Furthermore, the insights from the feature importance analysis could inform adaptive question-selection strategies. Specifically, future iterations of the inference algorithm could prioritize questions targeting highly unique or anatomically localized symptoms, especially in regions such as the Abdomen/Pelvis and Chest, which were identified as diagnostically challenging. Such targeted questioning could further enhance convergence speed and reliability under uncertain user input conditions. This is a matter of future work.

Overall, the discussion underscores both the robustness and the current boundaries of the system’s performance, providing a foundation for continued refinement and validation in realistic diagnostic contexts.

### Related work

#### Symptom checkers in human and veterinary medicine

Digital symptom checkers have become an established component of human healthcare, supported by an expanding body of systematic reviews, empirical evaluations, and policy-oriented studies published between 2021 and 2025. These works consistently document their widespread adoption for self-diagnosis, triage, and integration into healthcare systems across multiple countries (Chambers et al. 2019; El-Osta et al. 2025; Hammoud et al. 2024; Kopka et al. 2025; Pairon et al. 2023; Wallace et al. 2022; You et al. 2023). Systematic reviews confirm that symptom checkers are now widely deployed by both public and private providers to support patient self-assessment and early triage (Pairon et al. 2023; Wallace et al. 2022), while empirical studies highlight their growing institutional acceptance, with millions of monthly users and integration into national healthcare pathways such as NHS 111 and HealthDirect (Hammoud et al. 2024; Kopka et al. 2025). Despite continuing concerns about diagnostic precision and equity of access, the literature demonstrates consistent improvements in accuracy, user satisfaction, and system performance (Schmieding et al. 2025; Wallace et al. 2024), consolidating digital symptom checkers as core digital front doors to healthcare.

Despite the rapid progress of digital tools in human healthcare, few systems currently exist that are specifically designed and validated for animals. Recent reviews and empirical studies between 2023 and 2025 consistently highlight that digital decision-support systems, artificial intelligence (AI), and telemedicine applications in veterinary medicine remain underdeveloped, with limited standardization and validation frameworks compared to their human counterparts (Akinsulie et al. 2024; Xiao et al. 2025; Yusuf et al. 2024). Comprehensive analyses reveal that only a small fraction of clinical decision-support tools target animal health (six out of forty-one systems reviewed) underscoring a significant development gap (Xiao et al. 2025). Moreover, veterinary AI models often lack benchmark datasets, external validation, and regulatory oversight, resulting in poor generalizability and limited clinical readiness (Akinsulie et al. 2024; Xiao et al. 2025). While popular Web platforms such as CoVet AI and Vetscan Imagyst^TM^ demonstrate emerging examples of validated veterinary AI systems, the overall field remains in its early stages of digital transformation, with most applications still confined to pilot studies or proprietary implementations (CoVet AI 2025; Vetscan Imagyst^TM^ 2025).

The consensus across the literature is clear: veterinary digital health holds substantial potential but lags behind human healthcare in terms of validated, standardized, and clinically integrated systems. Unlike their human counterparts, most veterinary systems have not undergone rigorous diagnostic or triage validation, and only a handful of peer-reviewed evaluations have been published between 2020 and 2025 (Baran et al. 2025; Stans 2020; Xiao et al. 2025). The sole dedicated review of animal symptom checkers concluded that there is “little to no peer-reviewed research regarding the diagnostic accuracy” of these systems and emphasized their lack of probabilistic reasoning or benchmarking standards (Stans 2020). Broader analyses of veterinary AI applications confirm that digital diagnostic and triage models generally lack standardized datasets, external validation, and reproducibility, resulting in uncertain clinical reliability (Chiavaccini et al. 2024; Xiao et al. 2025). Although isolated validation efforts, such as Vet-AI’s automated triage tool reporting an internal accuracy of 81%, signal emerging progress, they remain unpublished in peer-reviewed literature and insufficient to establish confidence in the field (Improve Veterinary Practice Newsletter 2025; VetSurgeon News 2025). In contrast to human symptom checkers—whose diagnostic and triage performance has been systematically assessed across numerous studies—no comparable large-scale validation has yet been conducted for animals, leaving the diagnostic reliability of veterinary symptom checkers largely unknown.

Our study addresses the lack of validated veterinary symptom checkers identified in prior work. It provides a method validation of an expert-knowledge-based veterinary system, focusing on the correctness and internal consistency of its expert-derived knowledge database–a critical factor for practical reliability. Owing to the limited availability of real clinical datasets in veterinary research, we introduce a novel validation method based on synthetically generated test cases, thereby enabling systematic and reproducible exploration of the symptom–disease space. The results demonstrate high internal consistency, robustness, and computational efficiency, including a 100% convergence rate under ideal conditions, confirming the system’s technical soundness for preliminary triage. By establishing a validated foundation for future clinical and user-based evaluations, this work represents one of the first rigorous, reproducible validations of an animal symptom checker, marking an essential step toward aligning veterinary digital diagnostics with the evidence-based standards of human healthcare.

#### Symptom checker validation methods

Several methodological papers have been published to guide how symptom checkers, both in human and veterinary medicine, should be evaluated systematically and validly. These works converge on core methodological challenges, such as internal and external validity, representative vignette selection, and standardized reporting metrics. Together, they form a solid foundation for understanding how to design, benchmark, and compare digital and AI-based diagnostic systems.

Kopka and Feufel (2025) proposed an open-access framework for the consistent evaluation of digital health tools. Their work integrates best practices in case selection, reporting transparency, and performance metrics, addressing long-standing issues of methodological fragmentation. By aligning different evaluation traditions, they promote reproducibility and comparability across future studies, thereby establishing a cornerstone for rigorous symptom checker research. Kopka et al. (2023) also approached the evaluation problem from a psychometric viewpoint. They identified systematic biases in vignette-based benchmarking due to varying case difficulty and representativeness. To counter this, they introduced the Capability Comparison Score, a metric grounded in test theory that corrects for heterogeneity among vignettes, leading to fairer and more interpretable comparisons across systems. In his dissertation, Kopka (2025) extended this work through the lens of cognitive ergonomics and representative design. He argued for embedding real-world symptom distributions and probabilistic case modeling into evaluation protocols to better capture ecological validity. This contribution bridges theoretical principles with applied digital health evaluation, offering strategies to overcome the artificiality of synthetic vignette sets.

Painter et al. (2022) provided one of the earliest structured methodological guides for evaluating digital triage systems. It described detailed procedures for case generation, expert validation, blinded testing, and standardized accuracy reporting, e.g., top-1/top-3 accuracy and triage safety. Its proposed reporting framework later informed subsequent models by Kopka and colleagues, becoming a methodological reference point for the field.

El-Osta et al. (2022) investigated how the construction and validation of vignettes impact evaluation outcomes. The authors showed that inconsistencies between physician panels and diagnostic standards can substantially affect accuracy scores, underscoring the need for multi-expert consensus in reference data creation. Their findings highlighted the sensitivity of benchmarking results to design choices in vignette development.

Hammoud et al. (2024) conducted a large-scale empirical study applying a standardized framework across more than 200 vignettes validated by independent physicians . This work introduced novel quantitative metrics such as weighted diagnostic precision and made all materials publicly available, advancing reproducibility and transparency in digital health evaluation. It remains one of the most comprehensive empirical implementations of modern evaluation frameworks.

Finally, Schüz et al. (2022) provided a broader conceptual backdrop for these efforts. Grounded in Health Technology Assessment, it emphasizes ethical, usability, and population-level considerations, offering a transferable evaluation model applicable to AI-based symptom checkers. By integrating methodological rigor with regulatory and ethical perspectives, this framework supports the transition from research prototypes to clinically validated tools.

Collectively, these studies form a coherent methodological foundation for the evaluation of symptom checkers. They stress the importance of representativeness and realism in case design, psychometric fairness, transparency in data and metrics, and multi-expert validation to ensure credible benchmarking. This shared foundation enhances both the internal and external validity of symptom checker research, ensuring that evaluations are scientifically sound and comparable across contexts in both human and veterinary medicine.

Nearly all methodological papers on evaluating symptom checkers published to date are based on human medicine rather than veterinary medicine. The key methodological frameworks, such as introduced by Kopka et al. (2023); Kopka and Feufel (2025), and other benchmark studies, e.g., introduced by Hammoud et al. (2024); El-Osta et al. (2022), focus exclusively on human clinical contexts, employing human vignettes, physician reference standards, and healthcare triage pathways to assess diagnostic and triage accuracy. To the best of our knowledge, only one publication (Stans 2020) directly addresses diagnostic tools for animals, explicitly stating that “little to no peer-reviewed research has been published regarding animal symptom checkers” and that established human evaluation methods, such as vignette-based or confidence-based accuracy testing, have not yet been applied in veterinary contexts. Broader reviews on AI and digital decision support in veterinary medicine, e.g., (Chiavaccini et al. 2024), confirm this gap, noting that while veterinary AI systems for diagnostics and imaging are emerging, no dedicated methodological adaptation or standardized evaluation frameworks for veterinary symptom checkers currently exist.

Our study builds directly upon these methodological foundations by adopting and adapting rigorous validation frameworks originally developed for human symptom checkers to address the lack of standardized evaluation in the veterinary domain. The related work emphasizes representativeness, psychometric fairness, transparency, and multi-expert validation—principles mirrored in the present work’s design. Methodologically, our study aligns with efforts by Kopka and Feufel to standardize evaluation frameworks by defining clear performance metrics (Rank10, Rank25, Convergence, Top3) and integrating them into a single, reproducible composite difficulty score via PCA. Statistical rigor is maintained in our analysis. However, complementary to the rigid but *manual* vignette selection strategies of the related work, our approach proposes an *automatic* vignette generation using varying (probabilistic) answering strategies.

While recognizing the limitations of synthetic vignette validation, our study leverages synthetically generated test cases to systematically explore the symptom–disease space, addressing the scarcity of real-world veterinary datasets. Ultimately, this work represents a critical knowledge transfer: it adapts and complements the standardized, human-centric validation methodologies described in the related work to the veterinary context, providing one of the first rigorous methodological bridges between digital health validation in human and animal medicine.

## Conclusions

This study has validated the performance and structural properties of the expert-knowledge-based veterinary symptom checker, providing a detailed analysis of its robustness, efficiency, and the underlying factors influencing diagnostic convergence. The results demonstrate that the system is functionally stable, computationally efficient, and capable of producing clinically meaningful guidance under a variety of simulated user conditions. The validation study yielded several insights:

- *Demonstrated System Robustness and Efficiency (Q1 & Q2):* The symptom checker exhibited a high degree of robustness and computational efficiency. Under ideal simulated conditions, the system achieved an exceptionally high convergence ratio of 100.0%. The results confirm that the underlying inference rules and decision pathways are consistent and logically sound.
- *Suitability for Initial Triage:* The system demonstrated rapid convergence, with the mean disease rank reaching one after approximately 20 questions for the CORRECT strategy. Combined with extremely short response times (0.213–0.258 msec. per disease), this confirms the system’s technical suitability for initial triage and preliminary decision support in a clinical setting.
- *Handling Uncertainty:* Although performance decreased slightly under probabilistic answering strategies, the system remained relatively insensitive to uncertainty in user input. Non-converging cases were rare, and even when convergence criteria were not met, the correct disease typically appeared among the top-ranked suggestions (Ranks 1–6 for dogs; 1–4 for cats). These findings underline the system’s robustness against input variability and its potential utility as a reliable decision-support tool.
- *Structural Factors of Diagnostic Difficulty (Q3):* The study identified several structural determinants of diagnostic complexity:
  - The distribution of disease difficulty scores was skewed toward “easy” diseases, with a small subset of diagnostically challenging cases forming a long tail.
  - The number of symptoms and the uniqueness of symptom profiles emerged as the most influential predictors of disease difficulty.
  - Statistically significant differences in disease difficulty across anatomical regions were observed, indicating that diagnostic complexity varies systematically with disease location.

The results reported in this study are obtained under controlled simulation conditions with idealized answering strategies. In real-world settings, pet owners may provide incomplete, uncertain, or inaccurate responses, and symptoms may be interpreted differently, which can affect both convergence probability and speed. Consequently, the observed $100\%$ convergence under the CORRECT strategy represents an upper bound that is achievable only under idealized conditions and should not be interpreted as indicative of real-world performance. Also, we emphasize that the present work constitutes a method validation of the algorithmic behavior; clinical validation in real-world settings remains necessary.

Therefore, future research and development will focus on addressing the methodological limitations and enhancing the system’s clinical applicability:

- *Clinical and User Validation:* The present validation relied on synthetic test cases and automated answering strategies. To confirm real-world applicability, clinical trials and user studies are required and currently planned. These will involve empirical data from veterinary practice to assess system performance under authentic clinical and user conditions.
- *Knowledge Base Refinement:* Ongoing refinement efforts will target the small subset of diagnostically difficult diseases, particularly those characterized by low symptom uniqueness. Veterinary expert review will focus on enriching these diseases with more distinctive and discriminative symptom sets to enhance differentiation accuracy. This ongoing effort already shows success, as indicated by Fig. 10.
- *Adaptive Question-Selection Strategy:* Building on the feature importance analysis, future iterations of the inference engine should incorporate adaptive question-selection mechanisms. These will prioritize questions addressing highly unique or anatomically localized symptoms, particularly in diagnostically challenging regions such as Abdomen/Pelvis and Head/Neck. Such targeted questioning is expected to improve both convergence speed and diagnostic reliability under uncertain input conditions. The test system introduced in this paper will allow us to change and compare new strategies without regression.
- *Model Improvement:* The Random Forest regression model used to predict diagnostic difficulty achieved only moderate explanatory power. Future work should explore additional, more complex, or possibly more regulated predictors to better capture the determinants of diagnostic complexity, acknowledging that practical diagnostics (e.g., laboratory and imaging data) were intentionally excluded from the knowledge base.

Overall, this study establishes a foundation for the continued development of an intelligent, evidence-based, and clinically validated veterinary diagnostic support system. The findings provide both methodological insight and practical direction for advancing from simulation-based validation to real-world deployment. However, the proposed symptom checker is intended as a decision-support tool to assist users in structuring information and exploring possible conditions, and it is not a substitute for professional veterinary diagnosis.

## Data Availability

The knowledge database evaluated in this article is publicly available at: https://petsvetcheck.de/en.

## Footnotes

1 The statistics and all subsequent metrics are assessed on a snapshot of the knowledge database from Oct. 4th, 2025. While the database is by-and-large stable, it is constantly improved, so minor deviations between the documented and the current state may occur.

2 Principal Component Analysis (PCA) provides a data-driven approach to combine these correlated measures into a single composite score. By identifying the dominant direction of variance in the multidimensional metric space, PCA emphasizes the most informative combination of metrics while reducing noise and redundancy. The first principal component thus serves as a balanced, objective summary of overall diagnostic performance, allowing diseases to be compared along a common scale that reflects shared variation across all metrics rather than any single metric in isolation.

3 A violin plot visualizes the full distribution of a dataset by combining a boxplot and a density plot. Its width at each point shows how frequently values occur: wide parts mean many observations, narrow parts few. The central dashed line marks the median, the dotted lines the interquartile range. Compared to boxplots, violin plots reveal whether the data are skewed, multi-modal, or concentrated around certain values—making them ideal for comparing the spread and shape of score distributions across the answering strategies.
